# Menstrual Factors, Reproductive Factors and Lung Cancer Risk: A *Meta*-analysis

**DOI:** 10.3779/j.issn.1009-3419.2012.12.04

**Published:** 2012-12-20

**Authors:** Yue ZHANG, Zhihua YIN, Li SHEN, Yan WAN, Baosen ZHOU

**Affiliations:** 1 Department of Epidemiology, School of Public Health, China Medical University, Shenyang 110001, China; 2 Key Laboratory of Cancer Etiology and Intervention, Liaoning University, Shenyang 110036, China; 3 China Medical University Center For Evidence-based Medicine, Shenyang 110001, China

**Keywords:** Lung neoplasms, Age at menarche, Length of menstrual cycle, Female sex hormones

## Abstract

**Background and objective:**

Epidemiological studies have suggested that menstrual and reproductive factors may influence lung cancer risk, but the results are controversial. We therefore carried out a *meta*-analysis aiming to examine the associations of lung cancer in women with menstrual and reproductive factors.

**Methods:**

Relevant studies were searched from PubMed database, CNKI, WANFANG DATA and VIP INFORMATION up to January 2012, with no language restrictions. References listed from selected papers were also reviewed. We included studies that reported the estimates of relative risks (RRs) with 95% confidence intervals (CIs) for the association between menstrual and reproductive factors and lung cancer risk. The pooled RRs were calculated after the heterogeneity test with the software Stata 11, and publication bias and sensitivity were evaluated at the same time.

**Results:**

Twenty-five articles, representing 24 independent studies, were included in this *meta*-analysis. Older age at menarche in North America women (RR=0.83; 95%CI: 0.73-0.94) was associated with a significant decreased risk of lung cancer. Longer length of menstrual cycle was also associated with decreased lung cancer risk (RR=0.72; 95%CI: 0.57-0.90). Other exposures were not significantly associated.

**Conclusions:**

Our analysis provides evidence of the hypothesis that female sex hormones influence the risk of lung cancer in women, yet additional studies are warranted to extend this finding and to clarify the underlying mechanisms.

## Introduction

Lung cancer remains the leading cause of death from cancer among women in the United States^[1, 2]^. The incidence of lung cancer is increasing in women, in contrast to that seen in men. Interestingly, the proportion of lung cancer cases in females attributable to smoking is approximately half of that seen in males^[3]^. In addition, there is new evidence suggesting that estrogen receptors (ERs) ERα and ERβ have been detected on lung cancer cells in females^[4, 5]^. Laboratory evidence proves that estrogen acts as a promoter for lung adenocarcinoma in a mouse model based on genetic alterations that are relevant to the human condition^[6]^. These ﬁndings have suggested a hypothesis that female sex hormones, and consequently menstrual and reproductive factors, may play an important role in lung carcinogenesis.

Results from epidemiological studies, however, have been controversial. To evaluate the relationship between risk of lung cancer and menstrual and reproductive factors, we conducted a *meta*-analysis of these studies.

## Materials and methods

### Literature search

We attempted to report this *meta*-analysis in accordance with the *meta*-analysis of Observational Studies in Epidemiology guidelines^[7]^. We conducted a systematic literature search of the PubMed database, CNKI, WANFANG DATA and VIP INFORMATION through January 2012 by using the following search strategy: (menstrual factors OR reproductive factors) AND (lung cancer OR lung neoplasms OR pulmonary cancer OR pulmonary neoplasms), with no restrictions. Reference lists of the chosen papers were also reviewed for other potential articles that may have been missed in the database search. We did not contact the authors of the primary studies to request addition information.

### Study selection

Studies were included in our analysis if they met the following criteria: 1) the study design was a case-control study or a cohort study; 2) the endpoint of cohort study was incidence of lung cancer; 3) the risk estimate of lung cancer related to menstrual or reproductive factors and the corresponding 95% confidence intervals (CIs) were reported; studies without existing RRs were also eligible if the relevant data presented in the study we can use to calculate crude RRs instead; 4) of the studies with the same or overlapping population published in more than one study, we included the most recent ones.

### Data extraction and quality assessment

Two authors independently reviewed the articles and extracted the data, any disagreement was resolved by discussion. Information was recorded for each study as follows: last name of the first author, year of publication, study location, type of study design, length of follow-up (if applicable), total person-years of observation (if applicable), studied outcome, exposure variables and categories, number of lung cancer cases, number of controls, cohort size, age range (if applicable), menopausal status of the participants (if applicable), smoking status of the participants (if applicable), adjusted RR estimates from multivariable model with corresponding 95%CIs and statistical adjustment for potential confounders of interest.

Instead of providing aggregate scores, we assessed the quality of individual studies by recording the key components of study designs^[7]^, including characteristics of study populations, assessments of exposure and outcome and confounding variables controlled.

### Statistical analysis

In this reanalysis, the odds ratio (OR) and the hazard ratio (HR) were directly considered as RR. The categories of menstrual and reproductive exposure measures varied across studies, so we performed this *meta*-analysis of the comparison of the highest versus the lowest category in each study. In addition, exposures to OC use and HRT use were analyzed as dichotomous variables. Because some studies reported associations for different durations of OC use and HRT use in comparison with never use, we pooled those RRs to evaluate the total effect for ever versus never use.

Pooled risk estimates were calculated for exposure variables that were reported in at least five studies, which included age at menarche, length of menstrual cycle, number of pregnancies, parity (number of live births), age at first live birth, age at menopause, oral contraceptive (OC) use and hormone replacement therapy (HRT) use.

Other menstrual and reproductive variables reported in less than five studies included menopause status, other hormones, type of surgery, number of difficult labour, positive history of hysterectomy; miscarriage; spont; abortion, breast feeding, period length, menstrual regularity, quantity of menstrual flow, mental tension or pains related to menses, age at first use OC, age at first use HRT, years of menstruation, time since first use OC, time since last use OC, type of HRT, lifetime estrogen dose from HRT, intrauterine device use, age at last birth, estrogen plus progestin pills, calendar year of first use OC, HRT and number of menstrual cycles up to the reference date.

Heterogeneity test was performed with the use of *Q* statistic at the *P* < 0.05 level of significance^[8]^. We also calculated the *I*^2^ statistic, a quantitative measure of inconsistency across studies, which was classified as low (25%), moderate (50%) and high (50%)^[9]^. RRs from different studies were pooled using the fixed effect model and the random effect model based on the *Mantel-Haenszel* method and the Dersimonian and Laird method respectively. If there is no heterogeneity between studies, we used a fixed effect model; otherwise, we adopt the random effect model.

Subgroup analyses according to geographic region and study design were carried out to assess the potential impacts on the association. In addition, we conducted a sensitivity analysis to examine the influence of individual studies on the overall *meta*-analysis RR by sequentially omitting each one before pooling study-specific RRs.

Potential publication bias was assessed by visual inspection of *Begg's* funnel plots and formally by *Egger's* linear regression test^[10, 11]^. All statistical analyses were done using STATA version 11.0 software. A *P* value < 0.05 was considered statistically significant and all statistical tests were two-sided.

## Results

### Literature search

The literature search initially identified 926 publications from PubMed, CNKI, WANFANG and VIP INFORMATION; most were excluded because they were reviews or because the exposure or endpoint was not relevant to our analysis. After scanning, 33 were retrieved for further evaluation that also let to identification of 5 more articles from their collective references. Thus, 38 articles reported associations of at least one menstrual or reproductive variable with lung cancer risk. However, we excluded the articles by Fan *et al*^[12]^, Zheng *et al*^[13]^, Dorjgochoo *et al*^[14]^ and Liu *et al*^[15]^ because they provided insufficient data. Articles by Zhong *et al*^[16]^, Qin *et al*^[17]^, Yin *et al*^[18]^, and Fang *et al*^[19]^ were excluded because only point estimates and 95%CI were reported without categorical variables. We further excluded one study^[20]^ which studied on the survival of lung cancer. Two articles reported risk estimates based on the partially overlapping Chinese population^[22, 47]^, so we extracted data from Zheng *et al*^[22]^, the more recent reference, for all menstrual and reproductive variables. Two articles reported partially overlapping data from the Czech Women's Lung Cancer Study^[27, 48]^, however, Zatloukal *et al*^[48]^ reported the results separately by cell types of lung cancer, so we extracted all menstrual and reproductive data from Kubik *et al*^[27]^. Two articles based on the China Gansu Province population database overlapped^[31, 49]^, so data from the more recent reference were used^[31]^. One report analyzed unpublished data collected in a long-standing hospital-based case-control study^[24]^, which materials and methods were described in detail by Wynder *et al*^[25]^, so the information of study was extracted from Wynder *et al*^[25]^. Seow *et al*^[28]^ reported the results separately by smoking status, so we extracted two data sets from the study. Two articles reported partially overlapping data from the prospective Shanghai Women's Health Study^[38, 39]^, we extracted data from Chen *et al*^[39]^, the more recent reference, for all menstrual and reproductive variables except number of children and age at first live birth, which were only available from Weiss *et al*^[38]^. Two articles based on the population from Fujian reported overlapping results on age at menarche^[37, 43]^, so those results were extracted from the more recent reference^[43]^, other related variables were extracted from Chen *et al*^[37]^. Therefore, a total of 25 articles (18 written in English and 7 in Chinese), representing 24 independent studies, were included in this *meta*-analysis. A flow chart showing the study selection process is presented in **Fig 1**.

**1:**
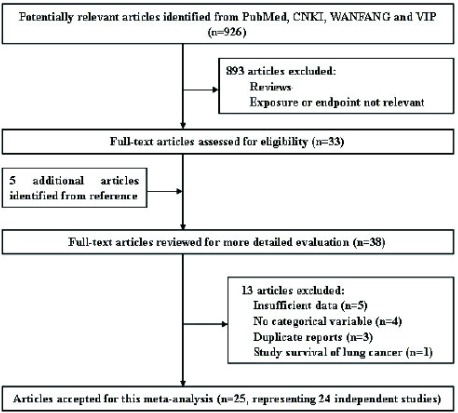
Flow chart of the literature search for studies of menstrual and reproductive factors and lung cancer risk

The characteristics of the included studies are presented in **Tab 1**. The 25 studies were published between 1988 and 2012. Of them, 13 studies had been carried out in Asia, 9 in North America and 3 in Europe. Eighteen studies reported case-control comparisons and 7 were analyses of cohorts.

**1:** Characteristics of observational studies addressing the association of lung cancer risk with menstrual and reproductive factors

Study	Region	Exposure(s) studied	Type of study (length of follow-up)	Cases/controls	Comparison group	Age range (menopausal status)	Smoking status	Type of cases	Adjustment for covariates
Wu *et al*, 1988^[21]^	United States	Age at menarche, menopause status, age at natural menopause, use of oral contraceptivesa, other hormones	Case-control	336/336	Population-based controls	30-75 (pre- and post- menopausal)	Ever smokers and current smokers	All adenocarcinoma	Pack-years off smoking, years since smoking stopped, and depth of inhalation
Zheng *et al*, 1988^[22]^	China	Length of menstrual cycle, age at menarche, age at menopause, surgery type, total number of menstrual cycles, total number of pregnancy, oral contraceptive, age at first full-term pregnancy and history of difficult labor	Case-control	672/753	Population-based controls	35-69 (pre- and post- menopausal)	Smokers and nonsmokers	Adenocaricinoma: 328; Squamous: 119; Small-cell undifferentiated: 34; Mixtures and other cell types: 61	Age, smoking and regularity of menstruation, age at menopause and age at menarche
Wu-Williams *et al*, 1990^[23]^	China	Age at menarche, number of children, age at natural menopause, hysterectomy, spontaneous abortion, pregnancy resulting in difficult labour, and use of oral contraceptives	Case-control	965/959	Population-based controls	(pre- and post- menopausal)	Smokers and nonsmokers	No data	Age, education, personal smoking and study area
Taioli *et al*, 1994^[24]^ and Wynder *et al*, 1977^[25]^	United States	Age at menarche, age at first pregnancy, number of full-term pregnancies, oral contraceptives, estrogen replacement, breast feeding (parous only), cycle length, period length, age at menopause, type of menopause	Case-control	180/303	Hospital-based controls	(pre- and post- menopausal)	Never, current and ex-smokers	No data	Smoking, age at diagnosis, years of education, BMI, menopausal status and type of menopause
Zhou *et al*, 2000^[26]^	China	Age at menarche, length of menstrual cycle, number of live births	Case-control	72/72	Population-based controls	35-69	Smokers and nonsmokers	All adenocarcinoma	Income, eye irritation from smoke, history of lung cancer
Kubik *et al*, 2002^[27]^	Czekh	Number of deliveries, number of miscarriages, age at menarche, cycle (repetition) of menses, duration of menstrual flow, quantity of menstrual flow, onset of menopause, mental tension or pains related to menses^a^.	Case-control	269/1, 079	Hospital-based controls	25-89 (pre and post menopausal)	Never, ex-smokers and current smokers	Adenocarcinoma: 79; small cell: 66; squamous cell:64; large cell: 16; bronchioloalveolar: 6; others: 25	Age, residence, education and pack-years of smoking
Seow *et al*, 2002^[28]^	Singapore	Number of livebirths, age at menarche, age at first childbirth, age at menopause, length of menstrual cycle^b^.	Case-control	303/765 (of whom 176 cases and 663 controls were lifetime nonsmokers)	Hospital-based controls	No data	Smokers and nonsmokers	Among smokers:adenocarcinama: 40; squamous cell: 38;small cell carcinomas: 19; others:30 Among lifetime nonsmokers: adenocarcinama: 126; squamous cell: 18;small cell carcinomas: 2; others: 30	Age, number of livebirths, family history of cancer. For smokers, adjusted additionally for duration and intensity. For nonsmokers, further adjustment for passive smoking did not materially affect estimates
Kreuzer *et al*, 2003^[29]^	Germany	Age at first menarche, length of menstrual cycle, age at first pregnancya, number of full-term pregnancies, menopausal status, age at natural menopause, use of oral contraceptives^a^, age at first use, duration of use, calendar year of first use, use of hormones, age at first use, duration of use^a^ and calendar year of first use.	Case-control	811/912	Population-based controls	< 76	No data	No data	Age, region, log (packyear+1), time since smoking cessation, and educational level and menopausal status
Xiang *et al*, 2003^[30]^	China	Age at menarche, menstrual cycle, OC use, number of live births	Case-control	149/128	Population-based controls	35-54	Nonsmokers	No data	Age, BMI and income
Brenner *et al*, 2004^[31]^	China	Age at menarche^a^, age at natural menopausea, average length of the menstrual cycle, average length of the menstrual flow, number of pregnancies, age at first live birth, number of live births, number of menstrual cycles up to the reference date.	Case-control	109/435	Population-based controls	No data	Smokers and nonsmokers	Adenocarcinama: 1; squamous cell: 14; small cell carcinomas: 16; others: 7	Age, prefecture. Further adjustment for socioeconomic status, active smoking, environment tobacco smoke among non-smokers, amount of coal, and previous pulmonary diseases
Liu *et al*, 2005^[32]^	Japan	Menopausal status, hormone use, breast feeding, age at menarche, age at menopause, years of menstruation, parity and age at first live birth.	Cohort (8-12 years)	153/44, 677	Participants in the JPHC Study	Cohort I:40-59 Cohort Ⅱ:40-69	Never-smokers	Adenocarcinomas: 118; others: 20; unknown: 17	Age, PHC area, and passive smoking during childhood or in the workplace
Elliott *et al*, 2006^[33]^	United Kingdom	Parity, oral contraception status, duration (ever) of oral contraception use, time since last use of oral contraception (ever users), time since first use of oral contraception (ever users), HRT status.	Nested case-control	162/486	Participants in the RCGP OCS	Mean age:29	Smokers and nonsmokers	No data	Smoking, social class and parity except where the variable itself is being examined
Kabat *et al*, 2007^[34]^	Canada	Parity^a^, age at first live birth^a^, age at menarche, oral contraceptive use, duration of OC use, HRT use and duration of HRT use	Cohort (16.4 years)	750/89, 835	Participants in the Canadian NBSS	40-59 (pre- and post menopausal)	Never, former smoker and current smoker	Squamous cell: 100; adenocarcinama: 355; small cell: 122; large cell: 49; other and mixed types: 102; missing: 22.	Parity, age at menarche, age at first birth, menopausal status, OC use, HRT use, BMI, education, smoking status, pack-years of smoking, study center and randomization group
Matsuo *et al*, 2007^[35]^	Japan	Fertile life, age at menarche, menopause, age at menopause, pregnancy, age at first parity	Case-control	435/2, 175	Hospital-based	18-79 (pre- and post menopausal)	Never, former	Non-small cell	Age
Schwartz *et al*, 2007 ^[36]^	United States	Age at menarche, menstrual cycles always or usually regular, length of menstrual cycle, age at menopause, years of menses, reason for menopause, age at first live birth, number of pregnancies, number of children, ever used OCs, duration of OC use, ever used HRT, type of HRT, duration of HRT use, lifetime estrogen dose from HRT.	Case-control	488/498	Population-based controls	18-74 (pre- and post menopausal)	Never, ex-smoker and current smoker	Non-small cell	Age at diagnosis/interview, race, pack-years, family history of lung cancer, current BMI, personal history of chronic obstructive lung disease, years exposed to passive smoke in the workplace, and education level
Chen *et al*, 2008^[37]^	China	Age at menarche and number of live births^a^.	Case-control	97/121	Population-based controls	No data	Smokers and nonsmokers	No data	Age and education level
Weiss *et al*, 2008^[38]^	China	Menopausal status, age at menarche, age at menopause, crude reproductive period ^a^, parity (no of livebirths) ^a^, age at first birth, intrauterine device use, OC use, HRT use	Cohort	220/71, 314	Participants in the prospective Shanghai Women's Health Study	40-70	Lifetime nonsmokers	Adenocarcinoma:78	Passive smoke exposure
Chen *et al*, 2009^[39]^	China	Age at menarche, regularity of menstruation, menopause status, age at menopause, years of estrogen exposure, number of pregnancies, OC use, age at first use OC, duration of use OC, HRT use, intrauterine device use.	Cohort (9.24)	271/72, 829	Participants in the prospective Shanghai Women's Health Study	40-70	Nonsmokers	No data	Age, education and income
Koushik *et al*, 2009^[40]^	Canada	Age at menarche, menopausal status, oophorectomy, menopause type, age at menopause, number of pregnancies, number of live births, age at first pregnancy, age at first live birth and lactation duration.	Case-control	422/577	Population-based controls	≥35	Never, former and current smoker.	Adenocarcinoma: 201; squamous cell: 83; small cell: 73; large cell: 37; other histology: 28	Age, respondent status, ethnic group, number of years of schooling, mean census tract family income, and smoking
Seow *et al*, 2009^[41]^	Singapore	Number of livebirths^a^, age at menarche, age at menopause, use of hormonal contraceptives and use of hormone replacement therapy	Cohort (9.6 years)	298/35, 298	Participants in the Singapore Chinese Health Study	45-74	Ever smokers and lifetime nonsmokers	No data	Age at interview, year of interview, dialect group, educational level, BMI, total vegetable intake, total fruit/juice intake, *β*-cryptoxanthin, total isothiocyanates, and (except for nonsmokers) duration of smoking, cigarettes per day, and number of years since quitting
Baik *et al*, 2010^[42]^	United States	Age at menopause, age at menarche, type of menopause, parity, age at first birth, PMH use, OCP use, PMH type and PMH duration.	Cohort (22 years)	1, 729/107, 171	Participants in the Nurses' Health Study	all postmen opausal	Never smokers, former smokers and current smokers	Adenocarcinoma: 706; squamous carcinoma: 253; small cell carcinoma: 264	Age at menopause, age at menarche, parity, type of menopause, PMH use, OC use, smoking status, age at start smoking, cigarettes per day, time since quitting, fruit/vegetable intake, BMI, and environmental smoking exposure
Lin *et al*, 2010^[43]^	China	Age at menarche^a^.	Case-control	208/208	Population-based controls	≥20	No data	Adenocarcinoma: 107; squamous carcinoma: 21; alveolar cell carcinoma: 20; undifferentiated carcinoma: 5; large cell carcinoma: 9; small cell carcinoma: 4; others: 42	BMI and education level
Meinhold *et al*, 2010^[44]^	United States	Age at menarche, menopausal status, number of live births ^a^, age at first birth, age at last birth, OC use, menopausal hormone therapy, estrogen plus progestin pills and estrogen pills.	Case-control	430/611	Population-based and hospital-based controls	No data	Never smokers, former smokers and current smokers	All nonsmall cell lung cancer	Age, education, smoking, number of smoking adults in household and current household income
Paulus *et al*, 2010^[45]^	United States	Parity, age at first birth	Case-control	1, 004/848	Hospital-based controls	≥18	No data	No data	Age, smoking status, pack-years of smoking, and years since quitting smoking
Brinton *et al*, 2011^[46]^	United States	Age at menarche ^a^, parity, number of births, age at first live birth among parous women, OC use, years of use of OC, age at natural menopause, age at surgical menopause, bilateral oophorectomy, age at surgical menopause, both ovaries intact, menopausal hormone use and years of use of menopausal hormones	Cohort	3, 512/185, 017	Participants in the NIH-AARP Diet and Health Study	50-71	No data	No data	Age at entry into cohort, race/ethnicity, education, BMI, emphysema, smoking status and dose, age at menarche, and type of and age at menopause
BMI, body mass index; JPHC, Japan Public Health Center-based Prospective Study; PHC, public health center; RCGP OCS: Royal College of General Practitioners'Oral Contraception Study; NBSS: National Breast Screening Study; OC: Oral Contraceptive; HRT: Hormone Replacement Therapy; PMH: postmenopausal hormone; NIH-AARP: National Institutes of Health- American Association of Retired Persons. ^a^ Statistically significant result. ^b^ Statistically significant result for lifetime nonsmokers. ^c^ Statistically significant result for squamous, small and large cell cancer.									

### Age at menarche

Nineteen studies examined the relationship between lung cancer risk and age at menarche^[21-24, 26-29, 31, 32, 34, 35, 39-44, 46]^. However, Zheng *et al*^[22]^, Wu-Williams *et al*^[23]^, Zhou *et al*^[26]^, Seow *et al*^[28]^ and Matsuo *et al*^[35]^ used the oldest age at menarche as the referent category, hence these 6 adjusted RRs could not be pooled with others comparing the highest versus the lowest categories, so we calculated crude RRs according to the number of cases and controls, then we used them instead. Furthermore, we excluded one study^[42]^ because it did not provide the required data.

Study-specific RRs for the oldest age at menarche as compared with the youngest age ranged from 0.345 to 3.158, and the pooled RR was 0.93 (95%CI: 0.79-1.10)(Fig 2). The RR for case-control study was 0.92 (95%CI: 0.71, 1.17), and the RR for cohort study was 0.96 (95%CI: 0.79-1.18). Between-study heterogeneity was high (*I*^2^=65.2%), but in subgroup analysis according to geographic region we found a significant decreased risk of lung cancer associated with older age at menarche in North America women (RR=0.83; 95%CI: 0.73-0.94).

**2:**
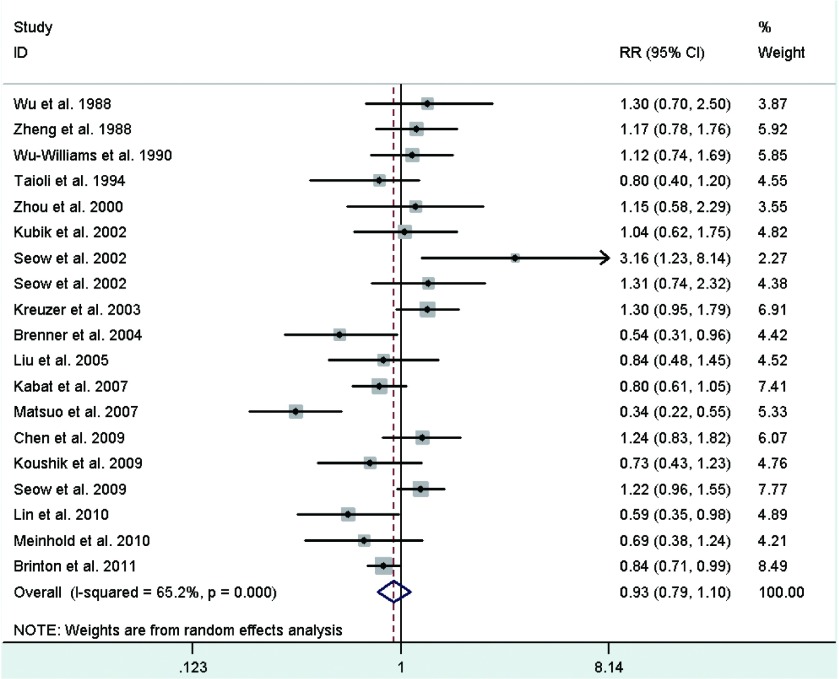
Random-effects *meta*-analysis of lung cancer relative risk (RR) associated with age at menarche (highest *vs* lowest category)

### Length of menstrual cycle

**Fig 3** represents a forest plot of the effect size distribution for the seven literatures^[22, 24, 26-29, 31]^ that studied on length of menstrual cycle. Zheng *et al*^[22]^ and Zhou *et al*^[26]^ used the longest length of menstrual cycle as the referent category, so we calculated and used crude RRs instead. Study-specific RRs for the longest versus the shortest length of menstrual cycle ranged from 0.46 to 0.91. The combined RR suggested a significant inverse association with a 28% decreased risk of lung cancer and low between-study heterogeneity.

**3:**
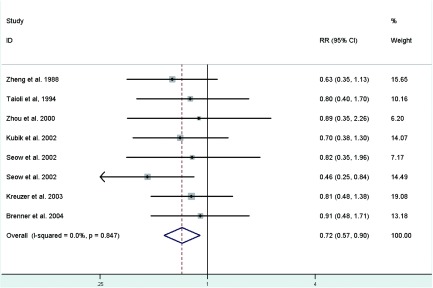
Fixed-effects *meta*-analysis of lung cancer RR associated with length of menstrual cycle (highest vs lowest category)

### Number of pregnancies

Associations of lung caner risk with number of pregnancies were suggested in seven studies^[24, 29, 31, 35, 36, 39, 40]^, among which two crude RRs^[35, 36]^ were calculated according to the number of cases and controls. Study-specific RRs for highest number of pregnancies as compared with the lowest ranged from 0.69 to 1.683. The pooled RR was 1.10 (95%CI: 0.91-1.34)(**Fig 4**), with low heterogeneity among the studies.

**4:**
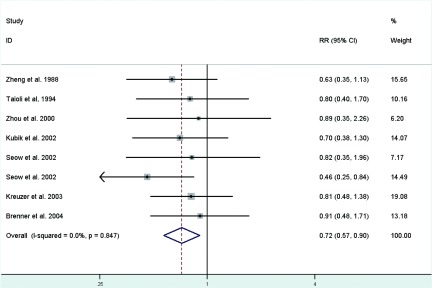
Fixed-effects *meta*-analysis of lung cancer RR associated with number of pregnancies (highest vs lowest category)

### Parity

Eighteen studies^[23, 26-28, 30-34, 36-38, 40-42, 44-46]^ provided information on parity, of which a crude RR^[36]^ was calculated based on the number of cases and controls, with study-specific RRs for highest number of live births in comparison with the lowest ranging from 0.39 to 4.744, and the summary RR was 0.91 (95%CI: 0.75-1.10; *I*^2^=75.8%)(**Fig 5**). In the eighteen studies, half studies^[27, 33, 34, 36, 38, 40, 41, 45, 46]^ used the nulliparous women as the reference group, the pooled RR was 0.93 (95%CI: 0.68-1.27; *I*^2^=82.4%)(**Fig 6**), whereas only three studies^[31, 42, 44]^ used a parous comparison group (1-2 children) so we did not estimate the risk, others were not clearly classified.

**5:**
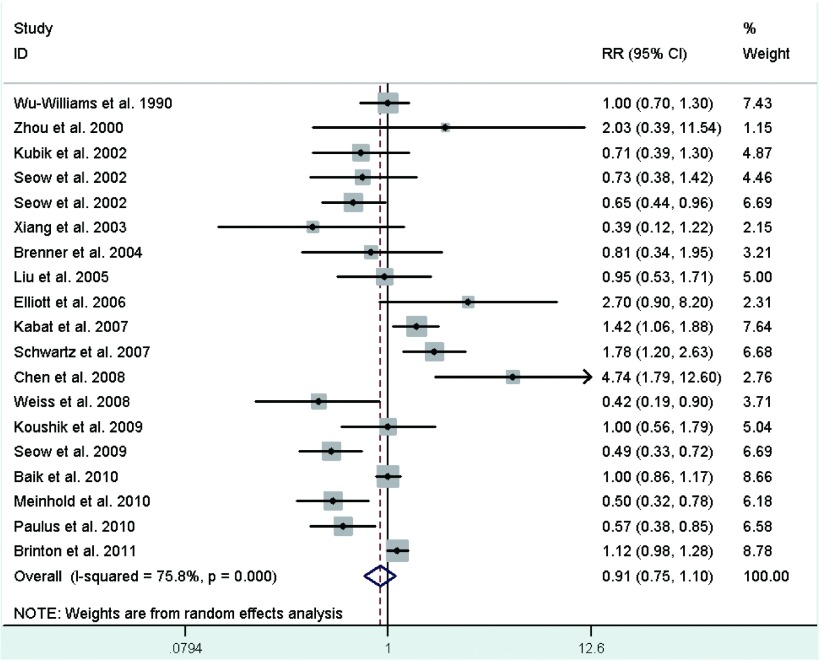
Random-effects *meta*-analysis of lung cancer RR associated with parity (highest *vs* lowest category)

**6:**
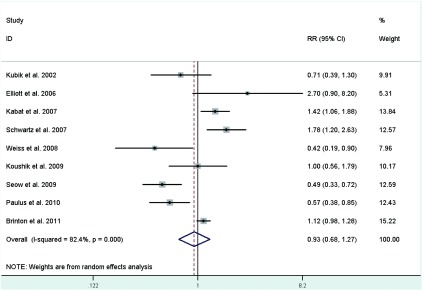
Random-effects *meta*-analysis of lung cancer RR associated with parity (highest *vs* nulliparous catrgory)

### Age at first live birth

Risk estimates for oldest versus youngest age at first live birth were reported in 11 studies^[28, 31, 32, 34, 35, 38, 40, 42, 44-46]^, including two crude RRs^[34, 42]^ instead, and ranged from 0.49 to 1.50. The combined RR was 1.03 (95%CI: 0.88-1.21)(**Fig 7**), with moderate heterogeneity across studies.

**7:**
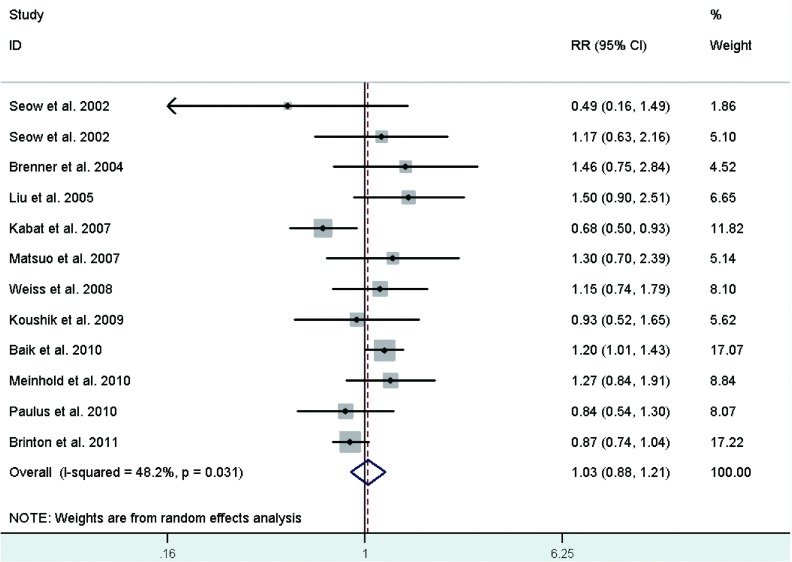
Random-effects *meta*-analysis of lung cancer RR associated with age at first live birth (highest *vs* lowest category)

### Age at menopause

Sixteen studies^[21-24, 27-29, 31, 32, 35, 39-42, 44, 46]^ reported the relationship between age at menopause and lung cancer risk, including five crude RRs^[24, 32, 35, 44, 46]^ calculated according to the number of cases and controls. However, two studies were excluded because one^[27]^ used not yet present as the referent category and the other^[42]^ did not provided required data. Study-specific RRs for the oldest age at menopause as compared with the youngest age ranged from 0.39 to 1.7. The pooled RR was 0.91 (95%CI: 0.70-1.17)(**Fig 8**), with high heterogeneity across studies.

**8:**
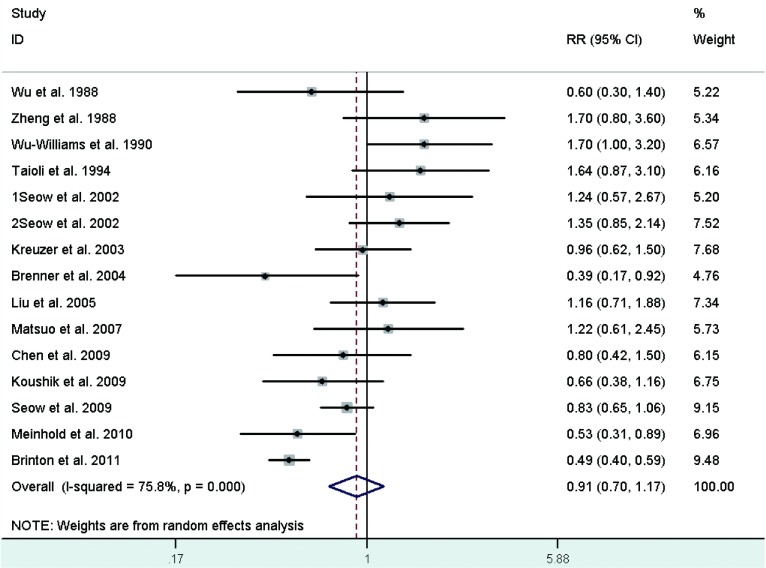
Random-effects *meta*-analysis of lung cancer RR associated with age at menopause (highest *vs* lowest category)

### OC use

Eight studies^[24, 29, 33, 34, 36, 39, 42, 44]^ reported the risk estimates for ever versus never OC use. What's more, the studies by Wu *et al*^[21]^ and Seow *et al*^[28]^ provided 2 RRs depending on duration of use, which we pooled to acquire overall RRs for ever use of 0.604 (95%CI: 0.273-1.336) for Wu *et al*^[21]^ and 0.854 (95%CI: 0.615-1.184) for Seow *et al*^[41]^. The pooled RR of lung cancer for ever users of OC as compared with never users was 0.97 (95%CI: 0.89-1.06)(**Fig 9**), with modern heterogeneity among the studies.

**9:**
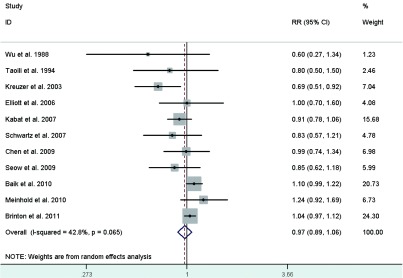
Random-effects *meta*-analysis of lung cancer RR associated with OC use (ever *vs* never)

### HRT use

Risk estimates for ever versus never HRT use were reported in eight studies^[24, 29, 33, 34, 36, 39, 41, 44]^, but one study^[29]^ was excluded because OC use was included in HRT use. In addition, the studies by Wu *et al*^[21]^, Baik *et al*^[42]^ and Brinton *et al*^[46]^ represented 2 RRs depending on duration of use, which we calculated to obtain overall RRs for ever use of 1.074 (95%CI: 0.749-1.539) for Wu *et al*^[21]^, 0.948 (95%CI: 0.859-1.046) for Baik *et al*^[42]^ and 0.934 (95%CI: 0.874-0.998) for Brinton *et al*^[46]^. The pooled RR of lung cancer for ever users versus never users of HRT was 0.99 (95%CI: 0.91-1.08)(**Fig 10**), with modern heterogeneity among the studies.

**10:**
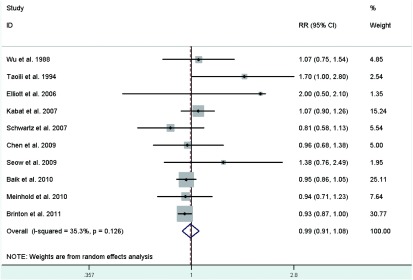
Random-effects *meta*-analysis of lung cancer RR associated with HRT use (ever *vs* never)

### Sensitivity analysis

A single study involved in the *meta*-analysis was omitted at a time to reflect the influence of the individual data set to the pooled RRs, and the corresponding combined RRs were not materially altered (**Fig 11**), suggesting that our results were stable and reliable.

**11:**
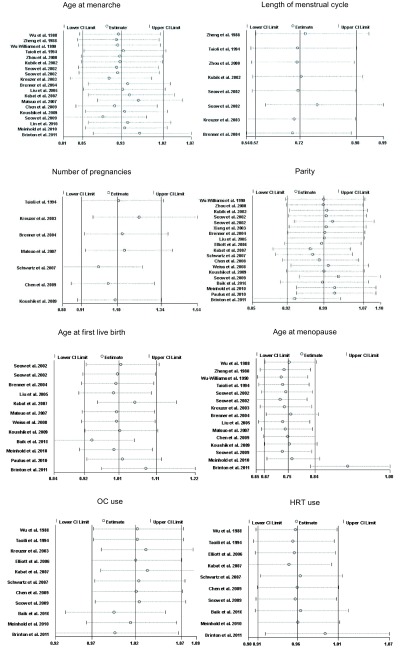
Results of the sensitivity analysis

### Publication bias

The *Begg's* funnel plot was conducted to assess the publication bias of studies (**Fig 12**). Then, the *Egger's* test was performed to provide statistical evidence of funnel plot symmetry. The P values for *Egger's* test were greater than 0.05 for all exposure variables with the exception of age at menopause (*P*=0.028; **Tab 2**) and HRT use (*P*=0.041; **Tab 2**), the results indicated publication bias for age at menopause and HRT use.

**12:**
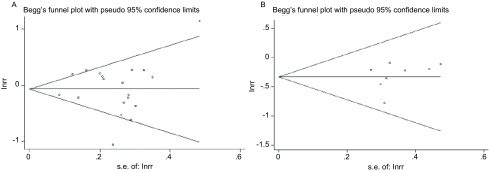
*Begg's* funnel plots with pseudo 95% CIs for lung cancer risk associated with age at menarche (A) and length of menstrual cycle (B)

**2:** Summary of *meta*-analysis results

Exposure	Exposure categories			Study design		Pooled RR for lung cancer (95%CI)	*P_Q_*	*I*^2^ (%)	*P_Egger's_*
	Highest (min to max)	Lowest (min to max)^a^		Case-control studies	Cohorts				
Age at menarche (y)	> 14 to 16-24	< 11 to ≤16		13	5	0.93 (0.79, 1.10)	< 0.001	65.2	0.889
Length of menstrual cycle (d)	> 28 to ≥34	< 26 to ≤30		7	0	0.72 (0.57, 0.90)b	0.847	0	0.448
Number of pregnancies	≥1 to ≥7	0 to 1-2		6	1	1.10 (0.91, 1.34)	0.239	24.9	0.689
Parity	> 2 to ≥7	0 to < 3		12	6	0.91 (0.75, 1.10)	0.008	75.8	0.409
Age at first live birth (y)	≥23 to ≥30	≤18 to < 26		6	5	1.03 (0.88, 1.21)	0.031	48.2	0.750
Age at menopause (y)	≥50 to 55-60	≤40 to < 50		11	5	0.91 (0.70, 1.17)	< 0.001	75.8	0.028^*^
OC use	Ever	Never		6	5	0.97 (0.89, 1.06)	0.065	42.8	0.062
HRT use	Ever	Never		5	5	0.99 (0.91, 1.08)	0.126	35.3	0.041^*^
*P_Q_, p* value from *Q* statistics; *P_Egger's_*, *p* value from *Egger's* test. ^a^ Reference category; ^b^Statistically significant.								

## Discussion

Several menstrual and reproductive factors have been suggested related to lung cancer risk, however, many of these results are inconsistent. There is a common point that women are more likely to be diagnosed with lung adenocarcinoma and non-small cell lung cancer (NSCLC). Our *meta*-analysis identified that decreased lung cancer risks were prone to present in women with longer length of menstrual cycle. We also found that age at menarche of North America women was inversely associated with lung cancer risk. Other six factors did not appear to be strongly associated with risk of this tumor. In summary, these findings support the hypothesis that estrogen exposure has an effect on the risk of lung cancer in women.

Shorter length of menstrual cycle indicated an overall increase in the period of unopposed estrogen exposure, and younger age at menarche implied more menstrual cycles over the lifetime and hence longer periods of estrogen exposure in total. Women who undergo shorter length of menstrual cycle and younger age at menarche may have an increased risk of lung cancer, possibly due to more cumulative exposure to endogenous estrogen, which may be involved in the etiology of this disease. There are several lines of evidence that estrogens may promote lung tumorigenesis: 1) estrogens can exert their biological effect through two ER subtypes, ERα and ERβ, particularly ERβ, which promotes estrogen-dependent growth of lung cancer cells^[50, 51]^; 2) hydroxylated estrogen metabolites can undergo redox cycling to generate free radicals, which cause DNA damage and lead to carcinogenic mutations^[52]^; 3) estrogens can directly stimulate the transcription of estrogen-responsive genes in the nucleus of lung cells and can also transactivate growth-factor-signaling pathways, such as the epidermal growth factor receptor (EGFR) pathway, which was involved in NSCLC growth, protection from apoptosis, and angiogenesis^[53-55]^; moreover, 4) estradiol (E2) can enhance the expression of midkine (MK) protein and E2 increased MK mRNA expression in lung adenocarcinoma cells. Both estrogen and MK can induce NSCLC epithelial-mesenchymal transition, which plays an important step in the migration of lung tumor cells^[56]^.

Furthermore, Henningson *et al* found that short length of menstrual cycle (< 27 days) were significantly more common with increasing number of variant A2 alleles^[57]^. The A2 allele was thought to enhance the transcriptional activity of the *CYP17* gene leading to elevated levels of estrogen^[58-60]^, which may increase the risk of lung cancer.

OC use and HRT use, as surrogates of exogenous sex hormonal exposure, seemed not to have a strong impact on lung cancer risk. This may suggest that endogenous and exogenous sex hormone play different roles in lung tumorigenesis, yet further researches using larger study populations are needed to confirm this assumption.

Heterogeneity is often a concern in a *meta*-analysis. Some evidence of heterogeneity was observed throughout our study. This was partially owing to the following facts: the studies we included focused on different types of design, most of them were case-control studies; studies we used were conducted in different geographic regions, mostly Asia and North America, where people share little in the field of genetic background, lifestyle, and lung cancer incidence; and the ranges of exposure variables in most studies were inconsistent. On this occasion, subgroup analysis was carried out to explain the heterogeneity. As a result, we found that differences in geographic region might contribute to the heterogeneity between studies.

*Egger's* test suggested little evidence of publication bias in our *meta*-analysis. We cannot preclude the possibility, as with any *meta*-analysis, that other unpublished studies may have been missed during our literature search. Meanwhile, we could hardly found the articles written in authors' mother tongue. Moreover, studies with null effects were less published than those with positive ones, which made it different for us to obtain.

Potential limitations of our *meta*-analysis should be considered. First, our analysis was limited by the inconsistent categorization of the exposure variables, especially those with more than two strata. However, all adjusted RRs were estimated on the basis of the highest versus the lowest category of the exposure variables, and the wide ranges of exposure variables probably reduced this bias. Second, residual confounders were always concerned in observational studies. Although we used the reported multivariable adjusted RRs where available, we still could not exclude the probability that other unmeasured factors have influenced the real relationship. Nonetheless, our study had a noteworthy strength. As individual study had insufficient statistical power, our *meta*-analysis of 24 studies involving a large number of participants enhanced the power to detect significant associations and provided more reliable estimates. Moreover, our results are consistent with the hypothesis that estrogen exposure may increase the risk of lung cancer in women, but the mechanisms involved are likely to be complex. It is clear that further studies, both mechanistic and epidemiologic, are warranted in this area. Our findings provide further evidence on the public health with respect to the lung cancer prevention in women.

## Conclusion

On balance, older age at menarche in North America women (RR=0.83; 95%CI: 0.73-0.94) was associated with a significant decreased risk of lung cancer. Longer length of menstrual cycle was also associated with decreased lung cancer risk (RR=0.72; 95%CI: 0.57-0.90). The other six exposures were not significantly associated. More investigations in large and well-designed studies are needed to extend these findings and to clarify the underlying mechanisms.

## Acknowledgements

This study was supported by grants No.81102194 from National Natural Science Foundation of China, No.LS2010168 from Liaoning Provincial Department of Education, and grant No.00726 from China Medical Board. The authors are most grateful to all the participants in this study.

## Conflict of interest

No competing financial interests exist for any of the authors.
